# Remodeling of the Dermal Extracellular Matrix in a Tissue-Engineered Psoriatic Skin Model by n-3 Polyunsaturated Fatty Acids

**DOI:** 10.3390/biomedicines10051078

**Published:** 2022-05-06

**Authors:** Mélissa Simard, Alexe Grenier, Geneviève Rioux, Andréa Tremblay, Isalie Blais, Nicolas Flamand, Roxane Pouliot

**Affiliations:** 1 Centre de Recherche en Organogénèse Expérimentale de l’Université Laval/LOEX, Axe Médecine Régénératrice, Centre de Recherche du CHU de Québec, Université Laval, Québec, QC G1J 1Z4, Canada; melissa.simard.6@ulaval.ca (M.S.); alexe.grenier.1@ulaval.ca (A.G.); genevieve.rioux.9@ulaval.ca (G.R.); andrea.tremblay.4@ulaval.ca (A.T.); isalie.blais.1@ulaval.ca (I.B.); 2 Faculté de Pharmacie, Université Laval, Québec, QC G1V 0A6, Canada; 3 Centre de Recherche de l’Institut Universitaire de Cardiologie et de Pneumologie de Québec, Département de Médecine, Faculté de Médecine, Université Laval, Québec, QC G1V 4G5, Canada; nicolas.flamand@criucpq.ulaval.ca; 4 Canada Excellence Research Chair on the Microbiome-Endocannabinoidome Axis in Metabolic Health (CERC-MEND), Université Laval, Québec, QC G1V 0A6, Canada

**Keywords:** psoriasis, inflammatory disease, n-3 PUFA, alpha-linolenic acid, bioactive lipid mediators, extracellular matrix, fibronectin, collagen, laminin

## Abstract

Psoriasis is an inflammatory skin disease mainly associated with an epidermal disorder. However, the involvement of the dermal extracellular matrix (ECM) composition in psoriasis is still poorly understood. This study aimed to investigate the expression of ECM components in psoriatic skin substitutes (PS^−^) compared with healthy skin substitutes (HS^−^), as well as the effect of an n-3 polyunsaturated fatty acid, namely α-linolenic acid (ALA), on the psoriatic dermal compartment (PS^ALA+^). Liquid chromatography tandem mass spectrometry analyses revealed that the lipidome of PS^−^ contained higher amounts of n-6 derived prostaglandins (PGE_2_) and lipoxygenase products (9-HODE and 15-HETE). ALA supplementation increased the levels of PGE_3_, 13-HOTrE, 15-HEPE, and 18-HEPE, and decreased the levels of PGE_2_, 15-HETE, and 9-HOPE compared with PS^−^, indicating that ALA modulates the dermal lipidome of psoriatic skin substitutes. Gene expression profiling showed that several genes encoding for different ECM proteins were overexpressed in PS^−^ compared with HS^−^, namely *COL1A1* (4.2-fold), *COL1A2* (3-fold), *COL3A1* (4.4-fold), *COL4A1* (2.3-fold), *COL4A2* (6.3-fold), *COL5A1* (3.3-fold), *COL5A2* (5.2-fold), and *COL5A3* (4.6-fold). Moreover, the expression of collagen IV (Col IV), collagen VII (Col VII), and laminin was found to be increased in PS^−^ compared with HS^−^, and to be restored with ALA (PS^ALA+^) according to immunofluorescence staining, while only the collagen I to collagen III ratio was altered according to dot blot analyses. Linear regression analysis revealed several positive correlations, including Col III with 14-HDHA levels, fibronectin with 12-HETE and 15-HETE levels, the dermo-epidermal junction Col IV with PGF_2__α_, 9-HODE, and 13-HODE levels, and laminin with levels of PGF_2__α_, 9-HODE, 13-HODE, 5-HETE, 12-HETE, and 15-HETE. These results suggest that the ECM plays an underestimated role in the pathogenesis of psoriasis and that ALA supplementation can regulate the ECM composition.

## 1. Introduction

Psoriasis is an inflammatory skin disease characterized by erythematosquamous plaques covered by white scales [1]. Psoriasis is primarily associated with the epidermis; however, psoriatic skin exhibits pathological changes in most, if not all, cutaneous cell types [2]. Psoriatic skin is characterized by hyperproliferative keratinocytes, whose presence leads to epidermal thickening (acanthosis) combined with the incomplete differentiation of the keratinocytes. Other defining histologic hallmarks of psoriasis include significant leukocyte infiltration as well as markedly increased vascularization [3]. Various treatments have been developed for the management of psoriasis, including topical, systemic, and biological agents [4,5]. Among the topical treatments, various combinations of topical ointments (such as vitamin D derivatives, topical corticosteroids, urea, and anthralin) have been proposed [6]. As shown recently, a formulation based on an alcohol-free foam with a predetermined association of a synthetic steroid/synthetic vitamin D3 analog appears to be a safe and effective way to treat mild to moderate forms of psoriasis, reducing the PASI score by up to 90% after 1 month of once-daily application of this treatment [7]. In recent years, a better understanding of the immunological basis of psoriasis has led to the development of several targeted biological therapies. Thus, the main therapeutic approaches focus on the modulation of T cell activity (alefacept, efalizumab, ustekinumab); the inhibition of the p19 subunit of the IL-23 cytokine, an actor that promotes the development and expansion of IL-17-producing T helper cells (guselkumab, risankizumab); IL-17 itself and its receptor (secukinumab, ixekizumab, brodalumab); and the inhibition of the tumor necrosis factor alpha (TNFα) cytokine (etanercept, infliximab, adalimumab) [8,9]. The complex etiology of psoriasis remains incompletely defined and mechanistic studies for effective therapeutic approaches are still ongoing [10]. Although the psoriatic epidermis has been comprehensively investigated, the role of dermal fibroblasts has been little studied, which may have contributed to an underestimation of the role of the latter in psoriasis. Indeed, the crosstalk between the dermis and the epidermis is essential for the maintenance of skin homeostasis [11].

The dermis is a connective tissue composed of fibroblasts that produce the extracellular matrix (ECM), mainly consisting of collagen fibers, elastic fibers, glycosaminoglycans, and proteoglycans [12]. The dermal collagen network that forms the principal skeleton of the ECM consists mainly of types I (Col I), III (Col III), and V (Col V) collagens and represents 70–80% of the skin’s weight [13]. Another important collagen network composes the basement membrane (BM) at the dermo-epidermal junction. This BM is formed from type IV collagen (Col IV), type VII collagen (Col VII), and laminins, with its function being to support the epidermis and preserve its integrity [14,15,16]. The ECM proteins and their receptors of the integrin family have been identified as important regulators of epidermal homeostasis, influencing the balance between cell renewal and differentiation [17]. The ECM composition of psoriatic skin is poorly documented. Among the ECM proteins, fibronectin has been the most associated with psoriasis, followed by laminin and type IV collagen. The expression of these proteins was found to increase in psoriasis [18,19,20,21,22].

Many pathological models are used to study complex skin diseases such as psoriasis in order to evaluate the innocuity and efficacy of potential new treatments [23]. Over the past decade, three-dimensional tissue-engineered human skin models produced with cells from psoriasis patients have been shown to have the most prominent features of psoriasis, including a hyperproliferative epidermis, abnormal keratinocyte differentiation, and altered gene expression [24,25,26,27]. Previously, our group explored the potential of n-3 polyunsaturated fatty acids (PUFAs) as a treatment for psoriasis in a tissue-engineered psoriatic skin model, focusing on the biological activity of n-3 PUFAs on psoriatic keratinocytes. We have shown that n-3 PUFAs decrease psoriatic keratinocyte proliferation and increase their differentiation, leading to the formation of a normal-looking epidermis. These effects were mediated by increased amounts of n-3 derived lipid mediators, decreased amounts of n-6-derived lipid mediators, and the activation of the extracellular signal-regulated kinase 1/2 (ERK1/2) signaling pathway [28]. In the present study, we expanded our analyses to study the expression of the dermal extracellular matrix in psoriasis as well as the impact of the n-3 fatty acid α-linolenic acid (ALA) on the psoriatic dermal compartment.

## 2. Materials and Methods

### 2.1. Cell Culture and Production of Tissue-Engineered Skin Substitutes

The Research Ethics Committee of the CHU de Québec-Université Laval approved the study and the volunteers signed a consent form in accordance with the Declaration of Helsinki and the guidelines of the Research Ethics Committee of the CHU de Québec-Université Laval. Healthy fibroblasts and keratinocytes were extracted from the breast reduction skin biopsies of three Caucasian women aged 18, 46, and 49 years old. Psoriatic fibroblasts and keratinocytes were extracted from 6 mm biopsy punches taken directly from the plaques of three psoriatic patients aged 46, 49, and 64 years old. The cells were extracted according to the method based on thermolysin, trypsin, and collagenase digestion described elsewhere [29].

Skin substitutes were produced according to the self-assembly method presented elsewhere [30,31]. Human fibroblasts (passage 6) were seeded in 6-well culture plates (1 × 10^4^ cells/cm^2^) with Dulbecco’s Modified Eagle’s (DME) medium (Gibco, Life Technologies, New York, NY, USA) supplemented with 10% Fetal Calf premium Serum (FCS) (Wisent Inc., St-Bruno, QC, Canada), 60 μg/mL penicillin (Sigma, Oakville, ON, Canada), 25 μg/mL gentamicin (Gemini Bio-Products, Sacramento, CA, USA), and 50 μg/mL ascorbic acid (Sigma, Oakville, ON, Canada). The 6-well plates were then incubated for 25 days. On the 25th day, two sheets of fibroblasts were superimposed and cultured for 3 days in a 100 mm Petri plate to form the dermal layer of the skin substitutes. The fusion of these sheets allowed the production of the dermal equivalents required for the seeding of primary human keratinocytes (passage 3, 1.2 × 10^6^ cells per dermal equivalent). The skin substitutes were kept in submerged conditions for 7 days in DME mixed with Ham’s F12 medium (3:1) (DMEH) (Gibco, Life Technologies, New York, NY, USA) including 5% FetalClone II serum (Galenova, Saint-Hyacinthe, QC, Canada), 5 μg/mL insulin, 0.4 μg/mL hydrocortisone, 10^−10^ M cholera toxin (Sigma, Oakville, ON, Canada), 10 ng/mL human epidermal growth factor (EGF) (Ango Inc, San Ramon, CA, USA), 60 μg/mL penicillin, 25 μg/mL gentamicin, and 50 μg/mL ascorbic acid. The skin substitutes with keratinocytes were then cultured at the air–liquid interface for 3 additional weeks in DMEH medium supplemented with 5% FetalClone II serum, 5 μg/mL insulin, 0.4 μg/mL hydrocortisone, 10^−10^ M cholera toxin, 60 μg/mL penicillin, 25 μg/mL gentamicin, and 50 μg/mL ascorbic acid.

Reconstructed substitutes were produced either with all culture media supplemented with ALA (HS^ALA+^ and PS^ALA+^) or with culture media supplemented with the corresponding volume of ethanol (0.003% EtOH) (HS^−^ and PS^−^). For n-3 PUFA supplementation, a stock solution was produced by dissolving ALA (Sigma, Oakville, ON, Canada) in 99% ethanol (Greenfield Global, Brampton, ON, Canada) [32,33]. Culture media were then supplemented so as to contain a final concentration of 10 μM ALA, a concentration selected according to our previous dose–response study [30]. The ALA solution was incorporated directly into the serum, which contained abundant bovine serum albumin, in order to increase its solubility in the complete culture medium. All cells were incubated at 37 °C under atmospheric conditions of 8% CO_2_. Culture media were changed three times a week.

### 2.2. Histological Analysis 

The biopsies were fixed in HistoChoice (AMRESCO, Inc., Solon, OH, USA) and encased in paraffin. Masson’s trichrome staining was executed on 5 micrometer-thick sections. Two substitutes for each of the three donors were analyzed (n = 6). The thickness of the dermis and the epidermis was measured on Masson’s trichrome-stained sections using ImageJ software (National Institutes of Health, USA, http://imagej.nih.gov/ij, accessed on 12 May 2021). Ten measurements in three different sections of each biopsy were made.

### 2.3. Immunofluorescence 

Tissue sections with a thickness of 5 µm were incubated for 10 min in cold acetone for effective fixation. Thereafter, the tissue sections were incubated for 45 min in a dark humidified chamber with the primary antibodies (Table S1) diluted in PBS containing 1% bovine serum albumin (BSA). After an adequate washout of the primary antibodies, the tissue sections were incubated for 30 min in a dark humidified chamber with the secondary antibodies (Table S1) diluted in PBS with 1% BSA. The slides were assembled in a mounting medium containing 4′-6′-diamidino-2-phenylindole (DAPI) (Fluoromount-G, SouthernBiotech, AL, USA), which stains the cell nucleus. A Zeiss microscope equipped with an AxioCam HR Rev3 camera (Oberkochen, Germany) was used to observe the tissues.

### 2.4. Profiling Gene Expression 

Total RNA was isolated from skin substitutes using the RNeasy Mini Kit (QIAGEN, Toronto, ON, Canada), and its quality was determined (2100 bioanalyzer, Agilent Technologies, Mississauga, ON, Canada) as described in the article by Rioux et al. [26]. The labeling of Cyanine 3-CTP labeled targets, their hybridization on a G4851A SurePrint G3 Human GE 8x60K array slide (Agilent Technologies, Santa-Clara, CA, USA), data acquisition, and analysis were all executed as indicated previously [26].

### 2.5. Protein Extract Preparation 

The dermis was removed mechanically from the epidermis using forceps and a scalpel. Tissues were transferred to 2 mL Safe-Lock Eppendorf tubes (ATS Scientific Inc., Burlington, ON, Canada) containing a 9 mm stainless steel ball and crushed using a Cryomill MM400 (Retsch^®^, Newtown, PA, USA). Samples were incubated in 250 µL of RIPA buffer with the protease inhibitor cOmplete (Roche, Mannheim, Germany) for 20 min on ice. The tubes were then centrifuged at 20,000× *g* for 20 min at 4 °C, after which the supernatants containing the proteins were collected and stored at −80 °C until their analysis. The proteins were quantified using a Pierce^TM^ BCA protein assay kit, following the recommendations of the manufacturer (Thermo Scientific, Rockford, IL, USA). 

### 2.6. Dot Blots 

A nitrocellulose membrane was placed in the Bio-Dot Apparatus (Bio-Rad, Mississauga, ON, Canada) and was rehydrated by injecting 100 μL of a tris-buffered solution (TBS) into each well. Total protein extract (5 µg or 10 µg) was loaded in the wells, and then each well was rinsed twice with 200 µL of TBS. The nitrocellulose membrane was removed from the device and rinsed in TBS with 0.1% Tween-10 solution (TBS-T). The antigenic sites were blocked for 1 h in TBS-T with a 5% powdered milk solution (Non-Fat Powdered Milk, Bio Basic, Markham, ON, Canada). The membranes were incubated for 1 h with the primary antibodies and for an additional hour with the secondary antibodies (Table S1). The proteins of interest were detected using an ECL Prime Western Blotting Detection Reagent (GE Healthcare, Little Chalfont, UK) and the Fusion F × 7 imager (MBI Lab Equipment, Kirkland, QC, Canada). Quantification of the dot blots was performed through densitometry using ImageJ (Wayne Rasband, National Institute of Health, USA).

### 2.7. LC-MS/MS 

The analysis of lipid mediators was performed as described in Simard et al. [28,33]. Briefly, the dermis was reduced to a fine powder (as described in Section 2.5), which was then suspended in 500 μL Tris-hydrochloride 50 mM (pH 7) and immediately denatured in one volume of cold methanol containing the internal standard (Table S2). Lipids were extracted using an acidified methanol–chloroform method as described elsewhere [34]. The extracted lipids were reconstituted in 50 μL of a liquid chromatography solvent (Solvent A and B, 50/50) and 40 μL was injected onto a reversed-phase HPLC column (Kinetex C8, 150 × 2.1 mm, 2.6 μm; Phenomenex, Torrance, CA, USA) in a LC-MS/MS system [35]. Solvent A was composed of water containing 0.05% acetic acid and 1 mM ammonium cation, while solvent B was composed of acetonitrile with water (95/5, *v*/*v*), 0.05% acetic acid, and 1 mM ammonium cation. Finally, lipids were quantified using calibration curves generated with pure standards in triplicates.

### 2.8. Statistics 

Data are expressed as mean ± standard deviation, except when stated otherwise. Statistical analyses were performed using ANOVAs followed by Tukey’s *post-hoc* tests. Only values of *p* < 0.05 were considered significant. All calculations were performed with Prism version 7 software (GraphPad Software, La Jolla, CA, USA).

## 3. Results

### 3.1. Characterization of the Skin Substitute Morphology

**Figure 1:**
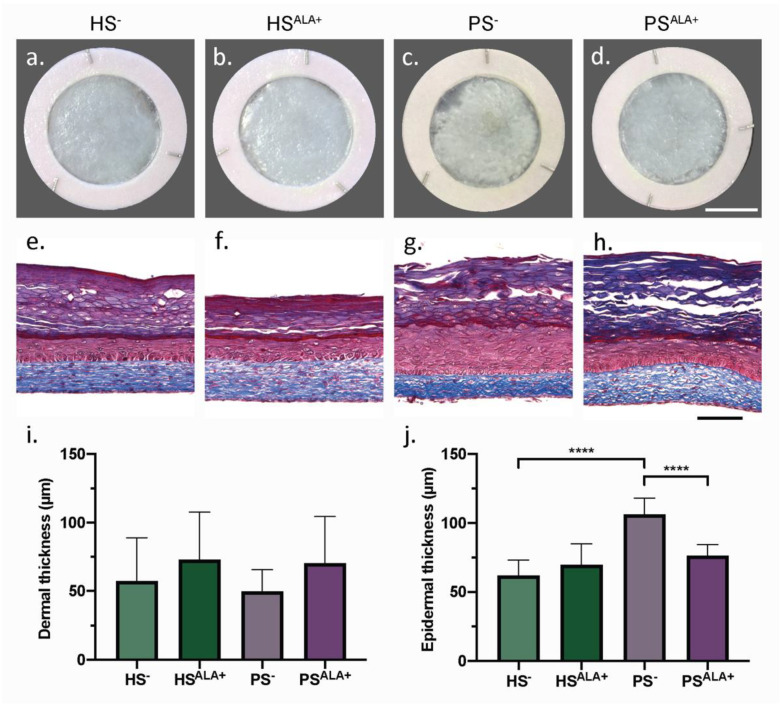
Biological activity of α-linolenic acid on skin substitute morphology. (**a**–**d**) Macroscopic appearance and (**e**–**h**) histological appearance after Masson’s trichrome staining of the skin substitutes. Scale bar: (**a**–**d**) 1 cm; (**e**–**h**) 100 µm. Thickness measurements of the dermis (**i**) and the epidermis (**j**) (N = 3 donors, n = 2 skin substitutes per donor). (**i**,**j**) Statistical significance was determined using one-way ANOVA followed by Tukey’s post-hoc test. **** *p* < 0.0001. Abbreviations: ALA—α-linolenic acid; HS—healthy substitute; PS—psoriatic substitute.

Healthy (HS) and psoriatic (PS) human skin substitutes were produced according to the self-assembly method with either culture media supplemented with ALA (HS^ALA+^ and PS^ALA+^) or unsupplemented (HS^−^ and PS^−^) in order to identify the effect of ALA on the cutaneous morphology of the skin substitutes (Figure 1). According to their macroscopic aspect, PS^−^ displayed a more disorganized epidermis than HS^−^ (Figure 1a,c). In addition, the epidermis was significantly thicker in PS^−^ than in HS^−^, showing that psoriatic keratinocytes preserved their hyperproliferative characteristic when cultivated in a 3D psoriatic skin model (Figure 1e,g,j). Interestingly, PS^ALA+^ had a more homogeneous epidermis and a significantly thinner epidermis than PS^−^, suggesting that treatment with ALA improved epidermal morphology notably by decreasing psoriatic keratinocyte proliferation (Figure 1c,d,g,h,j). The dermis, in which collagen fibers are stained in blue by Masson’s trichrome, was not significantly different between the various conditions (Figure 1e–h). The dermal thickness tended to increase after ALA supplementation in both HS^ALA+^ and PS^ALA+^, although this was not statistically significant (Figure 1i). 

### 3.2. Dual Effects of ALA Treatment on Lipid Mediator Levels in Psoriatic Skin Substitute Dermis 

**Figure 2:**
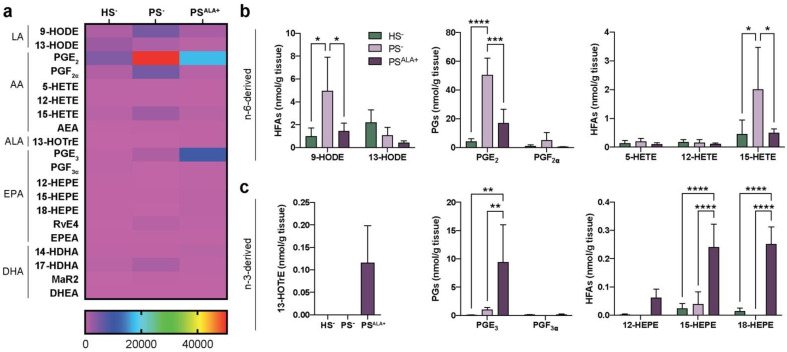
Levels of lipid mediators in the dermis. (**a**) Heatmap of bioactive lipid mediators found in the dermis of the skin substitutes as determined by targeted LC-MS/MS (N = 3). (**b**) n-6-derived and (**c**) n-3-derived bioactive lipid mediators from (**a**) that were the most differentially measured between HS^−^, PS^−^, and PS^ALA+^ dermis. Statistical significance was determined in (**b**,**c**) using two-way ANOVA followed by Tukey’s post-hoc test, with the exception of 13-HOTrE, for which statistical significance was determined using one-way ANOVA followed by Tukey’s post-hoc test. * *p* < 0.05; ** *p* < 0.01; *** *p* < 0.001; **** *p* < 0.0001. Abbreviations: AEA—*N*-arachidonoyl-ethanolamine; ALA—α-linolenic acid; DHEA—*N*-docosahexaenoyl-ethanolamine; EPEA—*N*-eicosapentaenoyl-ethanolamine; HEPE—hydroxyeicosapentaenoic acid; HETE—hydroxyeicosatetraenoic acid; HFA—hydroxy-fatty acid; HODE—hydroxyoctadecadienoic acid; HOTrE—hydroxyoctadecatrienoic acid; HS—healthy substitute; MaR—maresin; PG—prostaglandin; PS—psoriatic substitute; Rv—resolvin.

The levels of 20 bioactive lipid mediators were assayed using LC-MS/MS analyses to investigate the modulation of the lipidome of the skin substitute dermis by the ALA treatment (Table S3 and Figure 2). Significant alterations of lipid mediator levels were observed between PS^−^ and HS^−^ (Figure 2a). The most prominent lipid mediators found in the skin substitutes dermis were prostaglandins, followed by 15-LO metabolites such as 13-HODE, 15-HEPE, and 15-HETE (Figure 2a). The lipidome of the PS^−^ was significantly different from that of the HS^−^ (Figure 2a). Of note, significant increases were observed in the PS^−^ dermal levels of AA-derived metabolites, including prostaglandin E_2_ (PGE_2_), 9-HODE, and 15-HETE (Figure 2b). Treatment with ALA resulted in both increases in the n-3-derived lipid mediators and decreases in the n-6-derived lipid mediators (Figure 2a). As expected, the levels of ALA-derived 13-HOTrE were higher in PS^ALA+^ dermis, while it was not detected in PS^−^ dermis (Figure 2c). Additionally, the levels of EPA-derived PGE_3_, 15-HEPE, and 18-HEPE were also increased in PS^ALA+^ compared with both HS^−^ and PS^−^ (Figure 2b,c). In contrast, the levels of LA-derived 9-HODE, AA-derived PGE_2_, and 15-HETE were all decreased in PS^ALA+^ compared with PS^−^ (Figure 2b). These results show that ALA treatment modulates the dermal lipidome of psoriatic skin substitutes, leading to a profile enriched in bioactive lipid mediators associated with anti-inflammatory properties. 

### 3.3. Expression of the Extracellular Matrix Proteins in Healthy and Psoriatic Skin Substitutes

The expression of genes coding for the various proteins of the dermal extracellular matrix in healthy and psoriatic substitutes was studied using gene profiling on microarrays (Table 1). Genes with a linear signal higher than 100 were considered to be expressed in the skin substitutes, while genes with a linear signal under 100 were considered not to be detected under our experimental conditions. Thus, all collagen genes were expressed in both HS^−^ and PS^−^, with type I collagen genes (*COL1A1* and *COL1A2*) being the predominantly expressed collagen genes. Moreover, the expression of genes coding for types I (COL1), III (COL3), and V (COL5) collagens were all at least 2-fold higher in PS^−^ than in HS^−^. Indeed, enhanced expression of *COL1A1* (4.2-fold), *COL1A2* (3-fold), *COL3A1* (4.4-fold), *COL5A1* (3.3-fold), *COL5A2* (5.2-fold), and *COL5A3* (4.6-fold) was found in PS^−^. Interestingly, two of the type IV collagen (COL4) genes were overexpressed in PS^−^, namely *COL4A1* (2.3-fold) and *COL4A2* (6.3-fold). Finally, the expression of *COL7A1* was not different between HS^−^ and PS^−^.

### 3.4. Impact of ALA Treatment on the Expression of Extracellular Matrix Proteins in the Skin Substitutes 

**Figure 3:**
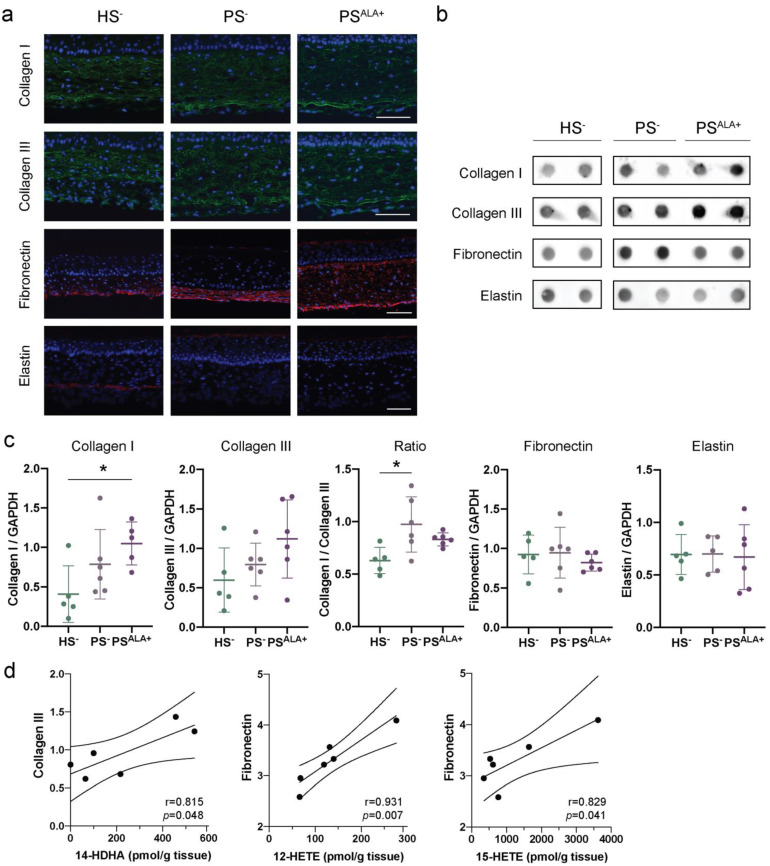
Impact of α-linolenic acid (ALA) on the levels of components of the dermal extracellular matrix in healthy and psoriatic skin substitutes. (**a**) Indirect immunofluorescence staining of collagen I (green), collagen III (green), fibronectin (red), and elastin (red). Nuclei were stained with DAPI (blue). Scale bar: 100 μm. (**b**) Dot blot analysis of collagen I, collagen III, fibronectin, and elastin. (**c**) Densitometric analysis of the dot blot from panel b. Statistical significance was determined using one-way ANOVA followed by Tukey’s post-hoc test. * *p* < 0.05. (**d**) Linear regression analyses assessing the correlation of ECM proteins to specific lipid mediators. The correlation coefficient was determined according to Pearson’s correlation coefficient (r), and the significance according to two-tailed test *p*-value (*p*). Values are means ± SD (N = 3 donors, n = 2 skin substitutes per donor). Abbreviations: ALA—α-linolenic acid; HETE—hydroxyeicosatetraenoic acid; HS—healthy substitute; PG—prostaglandin; PS—psoriatic substitute.

The expression of the different extracellular matrix proteins was studied using indirect immunofluorescence and dot blot analyses in order to confirm their presence in HS^−^ and PS^−^ and to evaluate whether ALA affects their expression (Figure 3). According to the immunofluorescence analyses, Col I and Col III were expressed uniformly throughout the reconstructed dermis both for HS^−^ and PS^−^ (Figure 3a). For their part, elastin and fibronectin were dispersed diffusely with a predominant localization at the bottom of the dermis (Figure 3a). The expression of ECM proteins in PS^−^ dermis was not found to be significantly altered compared with HS^−^ dermis under our experimental conditions. Indeed, although levels of Col I and Col III tended to be slightly higher in PS^−^ than in HS^−^, the difference was not significant (Figure 3b,c). Interestingly, the Col I to Col III ratio was significantly higher in PS^−^ than in HS^−^ (Figure 3c). Furthermore, elastin and fibronectin levels were not different in PS^−^ compared with HS^−^ (Figure 3b,c). Of note, high inter-individual variability was observed regarding the basal expression of extracellular matrix proteins. Regarding ALA treatment, the levels of Col I, Col III, elastin, and fibronectin were not statistically different between PS^ALA+^ and PS^−^, although levels of Col I and Col III tended to be higher in PS^ALA+^ than in PS^−^ (Figure 3b,c).

Linear regression analyses were performed to determine whether the expression of the ECM proteins correlated with the levels of bioactive lipid mediators (Figure 3d and Table 2). Interestingly, Col III expression was positively correlated with levels of n-3-derived lipid mediator 14-HDHA. In contrast, fibronectin levels were positively correlated with AA-derived (n-6) lipid mediator levels, specifically 12-HETE and 15-HETE (Figure 3d and Table 2). Additionally, the Col I/Col III ratio was positively correlated with the levels of RvE4 as well as the levels of most n-6 derived lipid mediators (Table 2 and Figure S1). Col I and elastin levels were not correlated to any lipid mediator levels (Table 2). Of note, PGF_2__α_ was detected in only two samples; therefore, linear regression analyses were not considered reliable for this metabolite.

### 3.5. Impact of ALA Supplementation on the Expression of the Dermo-Epidermal Junction Proteins in the Skin Substitutes

**Figure 4:**
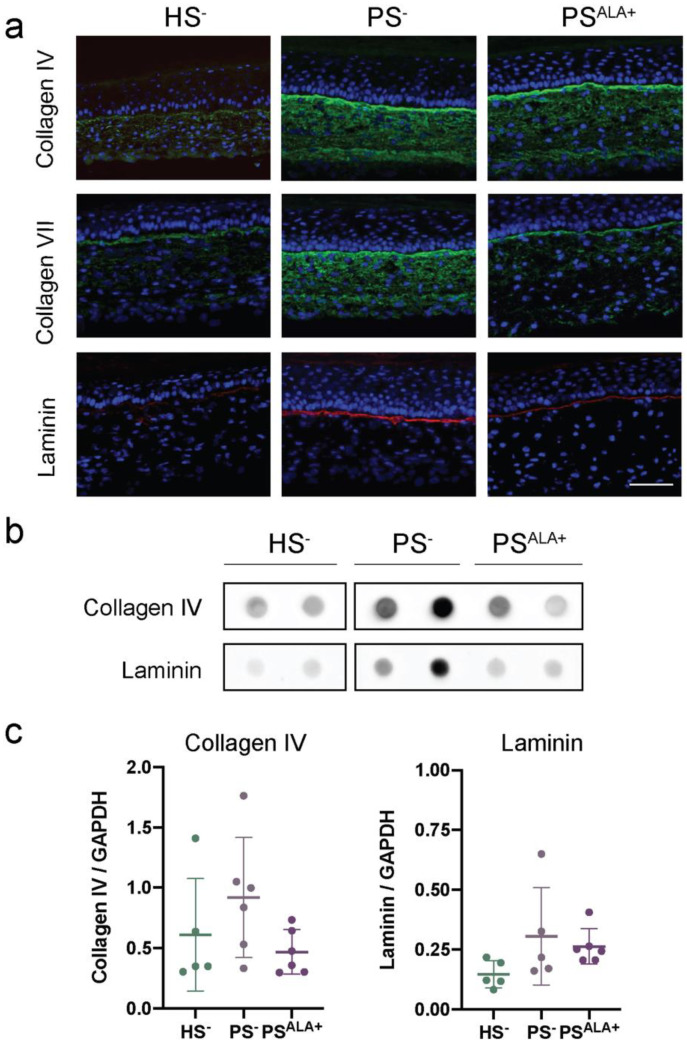
Impact of α-linolenic acid (ALA) supplementation on the levels of proteins at the dermo-epidermal junction of healthy and psoriatic skin substitutes. (**a**) Indirect immunofluorescence staining of collagen IV (green), collagen VII (green), and laminin (red). Nuclei were stained with DAPI (blue). Scale bar: 100 μm. (**b**) Dot blot analysis of collagen IV and laminin. (**c**) Densitometric analysis of the dot blot from panel (**b**) (N = 3 donors, n = 2 skin substitutes per donor). Statistical significance was determined using one-way ANOVA followed by Tukey’s post-hoc test. Abbreviations: ALA—α-linolenic acid; HODE—hydroxyoctadecadienoic acid; HS—healthy substitute; PS—psoriatic substitute.

The expression of the proteins at the dermo-epidermal junction was investigated using indirect immunofluorescence staining and dot blot analyses in order to evaluate whether their expression is disturbed in PS^−^ and whether ALA affects their expression (Figure 4). Based on the immunofluorescence analyses, all three proteins were expressed mainly at the dermo-epidermal junction (Figure 4a). However, while the expression of laminin was exclusively restricted to the dermo-epidermal junction, the diffuse expression of Col IV and Col VII was also observed throughout the rest of the dermis in the skin substitutes, and more markedly in the psoriatic substitutes (Figure 4a). Immunofluorescence analyses suggested that the levels of all three proteins were higher in PS^−^ compared with HS^−^, showing increased production of ECM proteins at the dermo-epidermal junction in psoriatic skin substitutes. However, the expression of ECM proteins was not found to be significantly altered in PS^−^ dermis compared with HS^−^ dermis under our experimental conditions according to dot blot analyses (Figure 4b,c). 

Based on immunofluorescence analyses, the levels of all three proteins were lower in PS^ALA+^ than in PS^−^, suggesting that ALA treatment had a beneficial impact on the expression of the proteins of the dermo-epidermal junction in PS^−^ (Figure 4a). Moreover, ALA seemed to reduce the presence of Col IV and Col VII in the dermis to a more restricted localization at the dermo-epidermal junction (Figure 4a). In contrast, the expression of Col IV and laminin was not found to be significantly decreased in PS^ALA+^ dermis compared with PS^−^ dermis under our experimental conditions according to dot blot analyses (Figure 4b,c). On the other hand, correlations were found between the expression of Col IV and laminin and the levels of n-6-derived lipid mediators in the psoriatic skin substitute dermis (Table 2 and Figure S2). Indeed, Col IV was positively correlated with the levels of PGF_2__α_, 9-HODE, and 13-HODE, while laminin was positively correlated with PGF_2__α_, 9-HODE, 13-HODE, 5-HETE, 12-HETE, and 15-HETE. 

## 4. Discussion

Psoriasis is an immune-driven skin disease mainly associated with an epidermal disorder including keratinocyte hyperproliferation and disturbed differentiation. The functional significance of the ECM in controlling epidermal stem cell fate has been investigated in many studies [36,37,38]. However, the implication of the ECM composition in psoriasis is still poorly understood. Moreover, while n-3 PUFAs were found to decrease psoriatic keratinocyte proliferation, improve psoriatic keratinocyte differentiation, and modulate epidermal protein expression [28,39,40], the impact of n-3 PUFAs on the dermal compartment of psoriatic skin was not studied. In the present study, ALA treatment regulated the ECM composition in psoriatic substitutes.

An increased expression of the genes encoding Col I, Col III, Col IV, and laminin was measured in psoriatic substitutes compared with healthy substitutes in our study. Other studies also seem to indicate an increase in the expression of collagen genes in psoriatic skin, as well as an increase in the levels of collagenase [41,42,43,44,45]. In contrast with our transcriptomic analyses, our dot blot analyses did not confirm altered ECM component expression at a protein level in psoriatic skin substitutes compared with healthy substitutes, thus suggesting a greater turnover of collagen in psoriatic skin substitutes. Accordingly, while the collagen protein levels reported in psoriatic skin vary between studies, all at least seem to agree on there being a greater turnover of collagen in psoriatic skin [46]. Of note, one study showed that collagen and elastin fibers tended to assemble in large bundles in native psoriatic skin, while smaller, more homogeneously spread fibers were found in healthy native skin [47]. Secondly, an increased type I/III collagen ratio was measured in psoriatic dermis compared to healthy dermis under our culture conditions. During wound healing, the type I/III collagen ratio is decreased in early granulated tissues, while the ratio is increased in mature scars [48]. Moreover, higher type I/III collagen ratios were found in disorders associated with loss of tissue compliance [49,50,51].

Subsequently, the unaltered levels of fibronectin, collagen IV, and laminin found in psoriatic substitutes compared with healthy substitutes show contrast with previous reports. The most studied ECM protein in psoriasis is fibronectin (more specifically, fibronectin-EDA), which was found to be significantly increased in native psoriatic and in imiquimod-mouse skin compared with their respective controls (healthy native skin, healthy mouse skin) [20,21,52]. Transforming growth factor-beta (TGF-β) together with fibronectin and 5β1 integrin (a fibronectin-specific receptor) were suggested to play a crucial role in the pathogenesis of psoriasis by influencing inflammation and keratinocyte hyperproliferation [53,54]. Furthermore, increased levels of Col IV were also found in native psoriatic skin and in imiquimod-treated mice [22,43]. Finally, most studies reported laminin disruption in psoriasis [20,21,52,55]. The expression of laminin in native psoriatic skin depends on the particular chains (α, β and γ) and isoforms measured. Toti and co-workers reported decreased laminin α2 chain and normal laminin α1, β1 and γ1 chains in psoriatic lesions [55], while Natsumi and co-workers reported increased laminin-332 (laminin α3, β3 and γ2; also known as laminin 5) and laminin-511 (laminin α5, β1 and γ1; also known as laminin 10) in psoriasis [22].

The impact of n-3 PUFAs on the expression of collagen has been studied widely, leading to a myriad of conclusions depending on the cell types, the model, the mode of administration, the various n-3 PUFAs, the tissues, and the diseases [56,57,58,59,60,61,62,63]. Based on the available data, the impact of PUFAs on collagen synthesis seems to be separated into two different responses, depending on whether the effects were assessed in a fibrotic tissue or in a wound-healing tissue. On the one hand, n-3 PUFA treatments seem to reduce collagen synthesis in fibrotic tissues [56,57,58,59]. Indeed, decreased collagen synthesis was measured after n-3 PUFA administration in a male Sprague–Dawley rat model of cholestasis, in a canine pacing model of atrial cardiomyopathy, in mice on a high-fat diet, and in mice with renal interstitial fibrosis [56,57,58,59,60,61]. On the other hand, most studies using EPA and DHA reported delayed wound healing and collagen synthesis [62,63]. In contrast, pro-resolving lipid mediators were shown to promote wound healing and, consequently, to stimulate collagen synthesis in wounded tissues [61].

The impact of n-3 PUFAs on the ECM proteins in psoriatic skin has not been directly investigated, and whether psoriatic dermis can be better associated with a fibrotic or a wounded tissue is not clear. Indeed, a few studies have compared psoriatic lesions to an everlasting wound that cannot be healed, while the enhanced collagen synthesis found in psoriatic skin is more closely related to fibrotic tissue conditions [64]. Interestingly, deeper analyses of the correlation between specific lipid mediators and ECM proteins seem to be the key to better understanding the divergent biological activities of n-3 and n-6 PUFAs in various conditions. In the present study, the levels of fibronectin, Col IV, and laminin were found to correlate with the levels of n-6-derived lipid mediators, while the levels of Col III correlated with the levels of n-3-derived lipid mediators. Fibronectin expression was also reported as being linked with the arachidonic cascade [65,66], or more specifically, with the levels of 12-HETE in vascular smooth muscle cells [67]. Moreover, stimulation with EPA and DHA was shown to have no impact on the expression of fibronectin in mesangial cell cultures [66], thus reinforcing the conclusion that fibronectin levels are regulated by n-6 metabolites and not n-3 metabolites. The overexpression of 12-LO (responsible for the conversion of AA into 12-HETE) in cardiac fibroblasts was associated with increased fibronectin levels and collagen synthesis [68]. However, other reports claimed that AA could trigger fibronectin degradation [69] and that it inhibited collagen synthesis [70]. Of note, various AA-derived lipid mediators seem to exert different and specific effects on the dermal ECM. For instance, PGE_2_ was shown to inhibit the mRNA expression of type I collagen α1 chain (*COL1A1*) in human dermal fibroblasts cultured in vitro [71]. Moreover, 5-HETE, 12-HETE, and LTB_4_ were found to be potent fibroblast chemoattractants [70]. In the present study, the levels of collagen III were found to correlate with the levels of PGF_3_ and 14-HDHA. To our knowledge, our study is the first to show such a link. It is interesting to note that collagen III synthesis is important during wound healing [72].

## 5. Conclusions

In conclusion, the mRNA expression of several ECM proteins and the levels of n-6-derived lipid mediators were higher in the psoriatic skin substitute dermis compared with healthy skin substitute dermis. These results seem to indicate an alteration of the dermal compartment in psoriatic skin. Supplementation of the culture medium with ALA modulated the dermal lipidome of psoriatic substitutes, resulting in increased levels of PGE_3_, 13-HOTrE, 15-HEPE, and 18-HEPE and decreased levels of PGE_2_, 15-HETE, and 9-HODE. Furthermore, the levels of bioactive lipid mediators were found to correlate with the levels of certain ECM proteins, showing that some lipid mediators may regulate the synthesis of the extracellular matrix in the dermis. Indeed, 14-HDHA would increase the expression of collagen III, while LA- and AA-derived lipid mediators would modulate the expression of fibronectin, collagen IV, and laminin.

## Figures and Tables

**Table 1:** Expression of genes encoding collagens in healthy and psoriatic skin substitutes.

Gene Symbol	Gene Name	Linear Signal HS^−^	Linear Signal PS^−^	Fold Change PS^−^/HS^−^
*COL1A1*	Type I collagen, alpha-1 chain	18,221	76,915	4.2 *
*COL1A2*	Type I collagen, alpha-2 chain	24,079	73,190	3.0 *
*COL3A1*	Type III collagen, alpha-1 chain	2759	12,201	4.4 *
*COL4A1*	Type IV collagen, alpha-1 chain	360	2229	2.3 *
*COL4A2*	Type IV collagen, alpha-2 chain	2279	14,503	6.3 *
*COL4A5*	Type IV collagen, alpha-5 chain	750	608	0.8
*COL4A6*	Type IV collagen, alpha-6 chain	505	332	0.7
*COL5A1*	Type V collagen, alpha-1 chain	1248	4116	3.3 *
*COL5A2*	Type V collagen, alpha-2 chain	1909	9909	5.2 *
*COL5A3*	Type V collagen, alpha-3 chain	100	460	4.6 *
*COL7A1*	Type VII collagen, alpha-1 chain	1118	1358	1.2
*LAMA1*	Laminin subunit alpha-1	105	118	1.1
*LAMA2*	Laminin subunit alpha-2	718	1586	2.2
*LAMA3*	Laminin subunit alpha-3	3650	1606	0.4
*LAMA4*	Laminin subunit alpha-4	187	225	1.2
*LAMA5*	Laminin subunit alpha-5	231	213	0.9
*LAMB1*	Laminin subunit beta-1	576	1357	2.4
*LAMB2*	Laminin subunit beta-2	2566	5487	2.1
*LAMB3*	Laminin subunit beta-3	9875	6013	0.6
*LAMB4*	Laminin subunit beta-4	119	120	1.0
*LAMC1*	Laminin subunit gamma-1	1669	4506	2.7
*LAMC2*	Laminin subunit gamma-2	1580	977	0.6
*LAMC3*	Laminin subunit gamma-3	59	56	0.9

* Considered different.

**Table 2:** Linear regression comparing ECM protein levels to the lipid mediator levels.

Lipid Mediators	Collagen I		Collagen III		Col I/Col III		Elastin		Fibronectin		Collagen IV		Laminin	
	r	*p*	r	*p*	r	*p*	r	*p*	r	*p*	r	*p*	r	*p*
PGE_3_	0.384	0.452	0.410	0.419	−0.259	0.621	−0.322	0.941	−0.149	0.777	−0.540	0.269	−0.149	0.534
PGF_3__α_	0.803	0.055	0.908	0.012	−0.360	0.483	−0.244	0.570	0.009	0.987	−0.279	0.593	0.009	0.641
13-HOTrE	0.585	0.223	0.807	0.052	−0.637	0.174	−0.191	0.875	−0.086	0.871	−0.314	0.544	−0.086	0.717
12-HEPE	0.562	0.246	0.668	0.147	−0.450	0.371	−0.339	0.952	−0.164	0.756	−0.537	0.272	−0.164	0.511
15-HEPE	0.518	0.293	0.717	0.109	−0.470	0.346	−0.097	0.808	−0.038	0.943	−0.309	0.551	−0.038	0.855
18-HEPE	0.345	0.503	0.483	0.332	−0.555	0.253	−0.343	0.681	−0.264	0.614	−0.632	0.178	−0.264	0.506
14-HDHA	0.639	0.172	0.815	0.048	−0.578	0.230	−0.251	0.973	−0.024	0.964	−0.449	0.371	−0.024	0.631
17-HDHA	0.256	0.625	−0.065	0.902	0.495	0.319	−0.225	0.098	0.450	0.371	−0.159	0.764	0.450	0.668
RvE4	−0.174	0.742	−0.471	0.346	0.843	0.035	0.239	0.294	0.526	0.283	0.396	0.436	0.526	0.649
MaR2	−0.402	0.430	−0.664	0.151	0.580	0.227	0.035	0.559	0.098	0.854	0.248	0.635	0.098	0.948
PGE_2_	−0.342	0.507	−0.553	0.255	0.911	0.011	0.529	0.572	0.556	0.252	0.681	0.136	0.556	0.281
PGF_2__α_	−0.483	0.332	−0.544	0.265	0.877	0.022	0.910	0.835	0.782	0.066	0.856	0.030	0.782	0.012
9-HODE	−0.424	0.402	−0.489	0.325	0.842	0.035	0.859	0.974	0.727	0.101	0.906	0.013	0.727	0.028
13-HODE	−0.406	0.424	−0.446	0.375	0.823	0.044	0.901	0.901	0.770	0.073	0.903	0.014	0.770	0.014
5-HETE	−0.351	0.495	−0.400	0.432	0.757	0.081	0.826	0.942	0.807	0.052	0.795	0.059	0.807	0.043
12-HETE	−0.116	0.827	−0.170	0.748	0.813	0.049	0.829	0.876	0.931	0.007	0.724	0.104	0.931	0.041
15-HETE	−0.387	0.448	−0.501	0.312	0.917	0.010	0.823	0.925	0.829	0.041	0.802	0.055	0.829	0.044

Values in green are significant.

## Data Availability

The gene expression data have been deposited in the National Center for Biotechnology and Information’s Gene Expression Omnibus (http://www.ncbi.nlm.nih.gov/geo/, accessed on 18 August 2021) and are accessible through Gene Expression Omnibus Series accession number GSE120464 (http://www.ncbi.nlm.nih.gov/geo/query/acc.cgi?acc¼GSE120464, accessed on 18 August 2021).

## Supplementary Materials

The following supporting information can be downloaded at https://www.mdpi.com/article/10.3390/biomedicines10051078/s1. Table S1: Antibodies used for immunofluorescence and dot blot analyses; Table S2: Specific mass transitions and retention times of the metabolites analyzed by LC-MS/MS; Table S3: Levels of bioactive lipids in skin substitutes after ALA supplementation. Figure S1: Linear regression analyses assessing the correlation of Col I/Col III proteins to specific lipid mediators. The correlation coefficient was determined according to Pearson’s correlation coefficient (r), and the significance according to two-tailed test *p*-value (p). Figure S2: Linear re-gression analyses assessing the correlation of laminin and collagen IV to specific lipid mediators. The correlation coefficient was determined according to Pearson’s correlation coefficient (r), and the significance according to two-tailed test *p*-value (p).
