# Supplementary material for: Remodeling of the Dermal Extracellular Matrix in a Tissue-Engineered Psoriatic Skin Model by n-3 Polyunsaturated Fatty Acids
Source: Biomedicines. 2022 May 6;10(5):1078. doi: 10.3390/biomedicines10051078 (PMC9138383; doi:10.3390/biomedicines10051078)
Supplement: Supplementary file 1 [file biomedicines-10-01078-s001.zip › biomedicines-1705864-SI.pdf]

Supplementary Materials

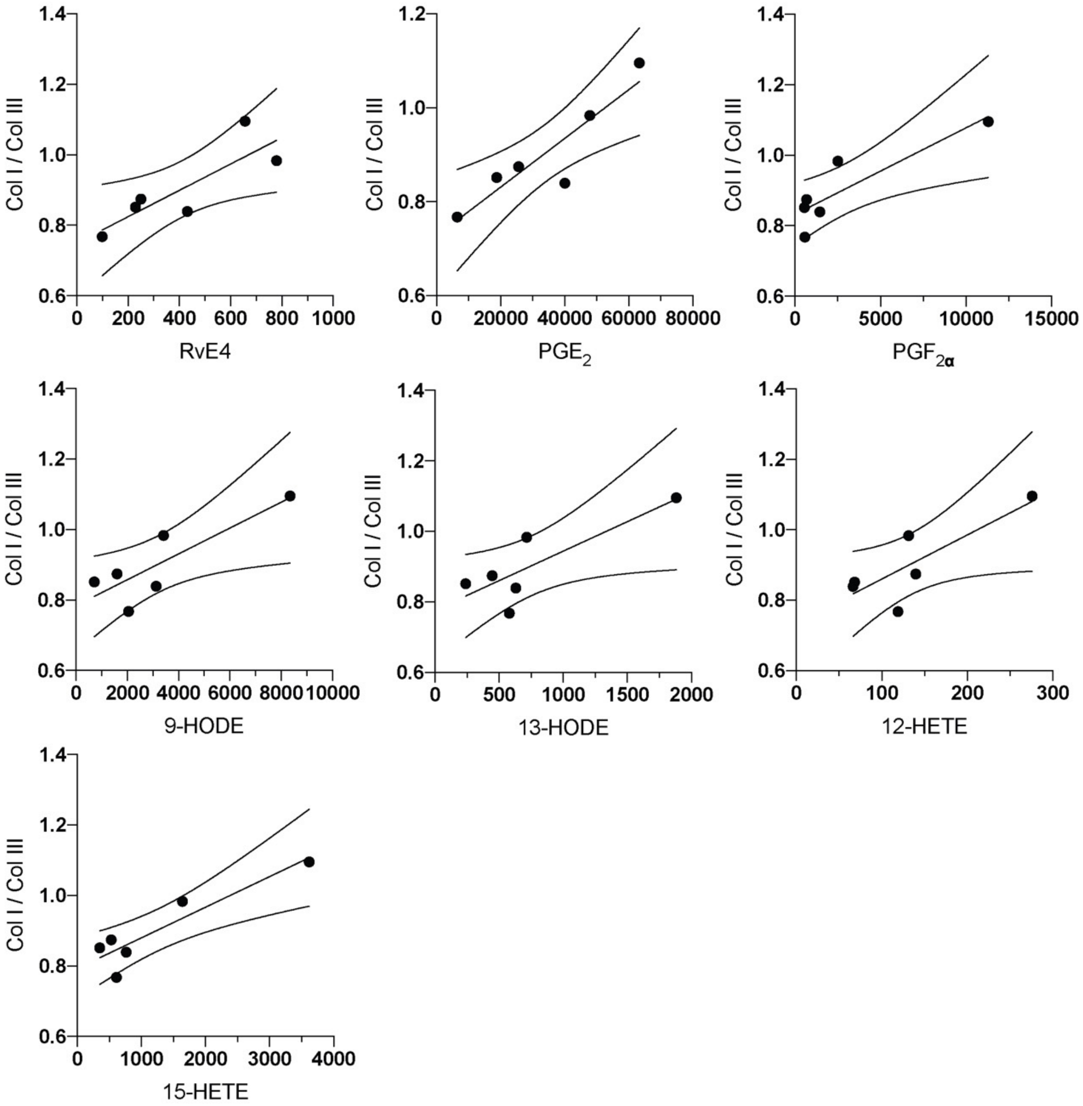

Figure S1. Linear regression analyses assessing the correlation of ECM proteins to specific lipid mediators. The correlation coefficient was determined according to Pearson's correlation coefficient (r), and the significance according to two-tailed test p-value (p).

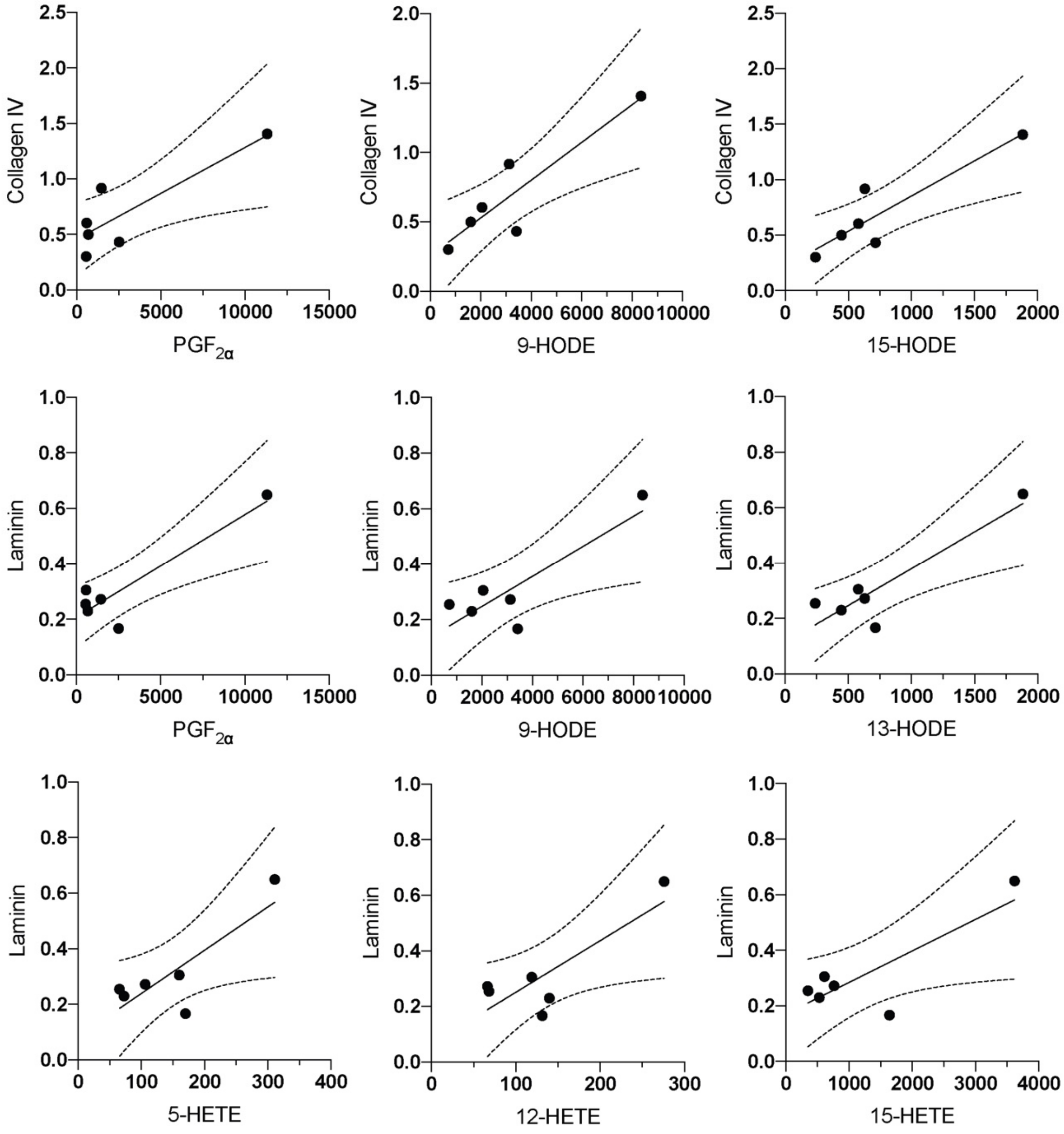

Figure S2. Linear regression analyses assessing the correlation of ECM proteins to specific lipid mediators. The correlation coefficient was determined according to Pearson's correlation coefficient (r), and the significance according to two-tailed test p-value (p).

Table S1. Antibodies used for immunofluorescence and dot blot analyses.

Antibody	Species	Manufacturer	Catalog number	Dilution IF	Dilution DB
Anti-Collagen I	Polyclonal rabbit	Cedarlane (Burlington, ON, Canada)	CL50111AP-1	1:100	1:2000
Anti-Collagen III	Polyclonal rabbit	Cedarlane (Burlington, ON, Canada)	CL50311AP-1	1:100	1:2000
Anti-Collagen IV	Polyclonal rabbit	Abcam (Cambridge, UK)	ab21295	1:100	1:1000
Anti-Fibronectin	Monoclonal mouse	ATCC (Manassas, VA, USA)	CRL-1606	1:100	1:500
Anti-Elastin	Polyclonal rabbit	Abcam (Cambridge, UK)	ab21610	1:30	1:1000
Anti-Laminin	Polyclonal rabbit	Abcam (Cambridge, UK)	ab11575	1:100	1:1000
Anti-GAPDH	Polyclonal mouse	BioLegend (San Diego, CA, USA)	649202	-	1:8000
Anti-rabbit Alexa 488	Polyclonal donkey	Life Technologies (Oakville, ON, Canada)	A21206	1:1600	-
Anti-rabbit Alexa 594	Polyclonal goat	Life Technologies (Oakville, ON, Canada)	A11012	1:1400	-
Anti-mouse Alexa 594	Polyclonal goat	Life Technologies (Oakville, ON, Canada)	A11005	1:1400	-
Anti-Rabbit Horseradish Peroxidase (HRP)	Polyclonal goat	Jackson ImmunoResearch (West Grove, PA, USA)	111-035-003	-	1:60000
Anti-Mouse Horseradish Peroxidase (HRP)	Polyclonal goat	Jackson ImmunoResearch (West Grove, PA, USA)	115-035-003	-	1:60000

Abbreviations: IF, immunofluorescence; DB, dot blot

Table S2. Specific mass transitions and retention times of the metabolites analyzed by LC-MS/MS.

Bioactive lipids	ISTD	Q1 → Q3	Retention time (min)	Detection limit (fmol)
9-HODE	13-HODE-d_4	295.30 → 171.35	9.48	5
13-HODE	13-HODE-d_4	295.50 → 195.30	9.48	5
PGE_2	PGE_2-d_4	351.20 → 271.15	5.07	5
PGF_{2a}	PGE_2-d_4	353.40 → 309.30	4.83	5
5-HETE	15-HETE-d_8	319.30 → 115.05	10.19	25
12-HETE	15-HETE-d_8	319.10 → 179.25	10.11	50
15-HETE	15-HETE-d_8	319.40 → 219.30	9.76	50
PGE_3	PGE_2-d_4	349.30 → 269.35	4.3	5
PGF_{3a}	PGE_2-d_4	351.30 → 193.30	4.08	5
12-HEPE	15-HETE-d_8	317.30 → 179.35	9.13	25
15-HEPE	15-HETE-d_8	317.40 → 219.25	9.04	50
18-HEPE	15-HETE-d_8	317.30 → 259.40	8.7	25
RvE4	RvD2-d_5	349.30 → 195.20	3.91	50
LTB_5	LTB_4-d_4	333.40 → 195.25	6.61	50
14-HDHA	15-HETE-d_8	343.50 → 281.30	10.59	50
17-HDHA	15-HETE-d_8	343.50 → 281.30	10.43	50
RvD5	RvD2-d_5	359.40 → 199.25	7.51	50
MaR2	RvD2-d_5	359.40 → 221.05	8.2	50

Table S3 Levels of bioactive lipids in skin substitutes after ALA supplementation.

Bioactive lipid mediators	Mean ±SD (pmol/g of tissue)			P-value	
	HS^-	PS^-	PS^{ALA+}	PS^- vs PS^{ALA+}	PS^- vs HS^-
**LA derivatives**					
9-HODE	1000.9 ± 723.9	4968.4 ± 2936.2	1456.4 ± 678.7	0.0341	0.0324
13-HODE	2205.1 ± 1089.5	1076.6 ± 698.9	421.9 ± 171.6	NS	NS
**AA derivatives**					
PGE_2	4137.4 ± 1984.2	50412.4 ± 11836.0	16988.0 ± 9699.4	0.0001	0.0001
$PGF_{2\alpha}$	956.9 ± 965.3	5090.0 ± 5407.7	604.9 ± 64.4	NS	NS
5-HETE	138.3 ± 91.6	195.6 ± 104.8	99.3 ± 52.5	NS	NS
12-HETE	177.3 ± 88.4	157.9 ± 107.3	109.1 ± 36.9	NS	NS
15-HETE	448.5 ± 500.4	2009.1 ± 1462.8	497.9 ± 132.7	0.0120	0.0198
AEA	0.9 ± 1.5	102.4 ± 18.1	89.9 ± 30.0	NS	0.0021
**ALA derivatives**					
13-HOTrE	ND	ND	115.5 ± 82.9	NS	NS
**EPA derivatives**					
PGE_3	82.8 ± 25.7	1001.0 ± 399.5	9395.1 ± 6651.4	0.0069	NS
$PGF_{3\alpha}$	119.6 ± 37.1	ND	137.9 ± 143.2	NS	NS
12-HEPE	2.5 ± 2.7	ND	61.8 ± 30.2	NS	NS
15-HEPE	24.0 ± 18.0	39.3 ± 43.4	240.7 ± 80.9	0.0001	NS
18-HEPE	13.9 ± 11.7	ND	251.7 ± 60.8	0.0001	NS
RvE4	ND	622.6 ± 176.8	192.7 ± 81.6	0.0081	0.0012
EPEA	2.2 ± 2.0	0.5 ± 0.3	7.1 ± 4.7	NS	NS
**DHA derivatives**					
14-HDHA	100.9 ± 77.0	55.1 ± 51.0	403.5 ± 166.9	NS	NS
17-HDHA	364.2 ± 282.1	1365.6 ± 1736.1	186.7 ± 99.3	NS	NS
MaR2	333.4 ± 112.9	40.3 ± 3.5	26.8 ± 4.9	NS	0.0031
DHEA	37.7 ± 40.4	4.4 ± 2.4	14.5 ± 10.0	NS	NS

Abbreviations: AA, arachidonic acid; ALA, α-linolenic acid; DGLA, dihomo-gamma-linolenic acid; DHA, docosahexaenoic acid; EPA, eicosapentaenoic acid; HEPE, hydroxyeicosapentaenoic acid; HETE, hydroperoxyeicosatetraenoic acid; HETrE, hydroxyeicosatrienoic acid; HDHA, hydroxydocosahexaenoic acid; HODE, hydroxyoctadecadienoic acid; LA, linoleic acid; LT, Leukotriene; ND, not detected; NS, not significant; PG, prostaglandin; Rv, Resolvin; SD, standard deviation.
